# Selection of Reliable Reference Genes for Gene Expression Analysis under Abiotic Stresses in the Desert Biomass Willow, *Salix psammophila*

**DOI:** 10.3389/fpls.2016.01505

**Published:** 2016-10-05

**Authors:** Jianbo Li, Huixia Jia, Xiaojiao Han, Jin Zhang, Pei Sun, Mengzhu Lu, Jianjun Hu

**Affiliations:** ^1^State Key Laboratory of Tree Genetics and Breeding, Key Laboratory of Tree Breeding and Cultivation of the State Forestry Administration, Research Institute of Forestry, Chinese Academy of ForestryBeijing, China; ^2^Collaborative Innovation Center of Sustainable Forestry in Southern China, Nanjing Forestry UniversityNanjing, China

**Keywords:** *Salix psammophila*, reference genes, quantitative real-time PCR, tissue types, abiotic stresses

## Abstract

*Salix psammophila* is a desert shrub willow that has extraordinary adaptation to abiotic stresses and plays an important role in maintaining local ecosystems. Moreover, *S. psammophila* is regarded as a promising biomass feedstock because of its high biomass yields and short rotation coppice cycle. However, few suitable reference genes (RGs) for quantitative real-time polymerase chain reaction (qRT-PCR) constrain the study on normalization of gene expression in *S. psammophila* until now. Here, we investigated the expression stabilities of 14 candidate RGs across tissue types and under four abiotic stress treatments, including heat, cold, salt, and drought treatments. After calculation of PCR efficiencies, three different software, NormFinder, geNorm, and BestKeeper were employed to analyze systematically the qRT-PCR data, and the outputs were merged by RankAggreg software. The optimal RGs selected for gene expression analysis were *EF1*α (*Elongation factor-1 alpha*) and *OTU* (*OTU-like cysteine protease family protein*) for different tissue types, *UBC* (*Ubiquitin-conjugating enzyme E2*) and *LTA4H* (*Leukotriene A-4 hydrolase homolog*) for heat treatment, *HIS* (*Histone superfamily protein H3*) and *ARF2* (*ADP-ribosylation factor 2*) for cold treatment, *OTU* and *ACT7* (*Actin 7*) for salt treatment, *UBC* and *LTA4H* for drought treatment. The expression of *UBC*, *ARF2*, and *VHAC* (*V-type proton ATPase subunit C*) varied the least across tissue types and under abiotic stresses. Furthermore, the relative genes expression profiles of one tissue-specific gene *WOX1a* (*WUSCHEL-related homeobox 1a*), and four stress-inducible genes, including *Hsf-A2* (*Heat shock transcription factors A2*), *CBF3* (*C-repeat binding factor 3*), *HKT1* (*High-Affinity K^+^ Transporter 1*), and *GST* (*Glutathione S-transferase*), were conducted to confirm the validity of the RGs in this study. These results provided an important RGs application guideline for gene expression characterization in *S. psammophila*.

## Introduction

*Salix psammophila* (*Salix*, Salicaceae), an important desert shrub willow, distributes in arid and semi-arid desert regions of Northern China, including Mu Us sandy land and Kubuqi desert in Inner Mongolia, Yulin in Shaanxi Province, Yanchi in Ningxia Province, and others. With extraordinary adaptation to environmental stresses (e.g., water deficit, extreme temperature and ion toxicity or deficiency) and strong ability in wind-breaking and sand-fixation, *S. psammophila* plays a significant role in maintaining local ecosystems (Bao and Zhang, 2012; Huang et al., 2015). Moreover, as a promising biomass feedstock for biofuels and bioenergy, *S. psammophila* has been applied in paper making, production of bio-based chemicals and hydrochar, such as carboxylic acids, phenolic derivatives, furan compounds, solid fuel, and adsorbents (Li et al., 2013; Yang et al., 2014). Owing to its remarkable advantages and value, the interest in the study of discovery of stress- and development-related genes on *S. psammophila* is stimulated for some researches.

Understanding the gene expression patterns will be of great help in the systematic elucidation the gene mechanism in complex regulatory networks. Many methods are used to assess gene expression level. Thereinto, quantitative real-time polymerase chain reaction (qRT-PCR) is the most prevalent method in detecting gene expression level with certain advantages of speed, high sensitivity, convenience, benefits, reproducibility, and accuracy (Bustin, 2002; Bustin et al., 2005; Nolan et al., 2006). However, the accuracy of experimental data obtained by qRT-PCR always was affected by many variable factors, such as RNA quality, reverse transcription efficiency (Bustin, 2002; Udvardi et al., 2008). In order to minimize the experimental errors, reference gene (RG) is used to normalize the experimental data from qRT-PCR (Radonić et al., 2004), so the accuracy of experimental results of qRT-PCR often depends on selected RGs which are appropriate or not.

An ideal RG should be stable expressed in different tissue types, development stages and under different experimental conditions. Then, housekeeping genes involved in basic cellular processes are used as representative of the traditional internal RGs in many studies, such as *ACT* (*Actin*), *TUB* (*Tubulin*), *18S* (*18S ribosomal RNA*), *UBC* (*Ubiquitin-conjugating enzyme*), *EF1*α (*Elongation factor-1 alpha*) (Fiume and Fletcher, 2012; Mousavi et al., 2013; Sakuraba et al., 2014; Tian H. et al., 2015; Xu et al., 2015; Liu et al., 2016). However, many studies also show that housekeeping genes have been not stably expressed under any tissues and all experimental conditions (Schmittgen and Zakrajsek, 2000; Jain et al., 2006; Hong et al., 2008; Wang et al., 2014; Xu et al., 2016). For example, the expression of *18S rRNA* was stable in long-duration salt treatment, but it was variable in drought treatment in *Populus euphratica* (Wang et al., 2014). *EF1*α was an ideal RG in different tissues and abiotic stresses, but it was not suitable in the different stages and hormone treatment in *Brachypodium distachyon* (Hong et al., 2008). The expression of *ACT2/7* and *ACT11* were unstable under stress treatments in soybean (Yim et al., 2015). *UBC2* was also unstable in abiotic stress, organ and tissue in watermelon (Kong et al., 2014a).

Recent research found that many new RGs were better than traditional genes as ideal genes, e.g., *PP2A* (*protein phosphatase 2A*) in buffalo grass (Li et al., 2014) and hybrid roses (Klie and Debener, 2011), *CUL1* (*CULLIN 1*) and *APT3* (*ADENINE PHOSPHORIBOSYL TRANSFERASE 3*) in *Marchantia polymorpha* (Saint-Marcoux et al., 2015), *Bic-C2*, *F-box protein 2* and *VPS-like* in soybean (Yim et al., 2015), *RPL* (*ribosomal protein L*) and *RPS15* (*cytosolic ribosomal protein S15*) in melon fruits (Kong et al., 2016). Therefore, it is important to select suitable RGs under different conditions in various species.

Many studies of RGs expression have been reported in *Arabidopsis thaliana* (Remans et al., 2008), rice (Jain et al., 2006), potato (Nicot et al., 2005), tomato (Expósito-Rodríguez et al., 2008), watermelon (Kong et al., 2014a), melon (Kong et al., 2014b), poplar (Wang et al., 2014), sorghum (Reddy et al., 2016), tea plant (Wu et al., 2016), tree peony (Li et al., 2016), *Eucalyptus* (Oliveira et al., 2012), and so on. Nevertheless, no study on validating of RGs has been conducted in *Salix* genus until now. To data, *Actin* and *Tubulin* are the most widely used as RGs to analyze gene expression profiling in various tissues and under different abiotic stress in *Salix matsudana Koidz* (Liu M. et al., 2014; Song et al., 2015; Yang et al., 2015) and *Salix suchowensis* (Zhang et al., 2015). However, recent studies had demonstrated that both of *ACT* and *TUB* were poorly used as RG for qRT-PCR in developmental stages of leaf and hormone stimuli in tea plant (Wu et al., 2016), and *ACT* was not the optimal RG in melon fruits (Kong et al., 2016). *TUB* was unstable in different tissues and abiotic stress in sorghum (Reddy et al., 2016). So it is doubtful to use *ACT* or *TUB* as RG in the *Salix* study. Recently, with the completion of whole-genome sequence of *Salix purpurea* and *Salix suchowensis*, the researches of gene function in *Salix* are rapidly launched. Thus, the systematic exploration of RGs under different experimental conditions for accurate transcript normalization is important and requisite for the future research in *Salix* genus.

In this study, 14 endogenous genes were selected as candidate RGs, and their expression stabilities were detected across tissue types and under different abiotic stress treatments in *S. psammophila*. Three software (NormFinder, geNorm, and BestKeeper) were employed to analyze systematically the qRT-PCR data, and the outputs were merged using RankAggreg software. In order to validate the suitability of these RGs, the relative genes expression profiles of one tissue-specific gene, *WOX1a* (*WUSCHEL-related homeobox 1*) (Nakata et al., 2012; Liu B. et al., 2014) and four stress-inducible genes, *Hsf-A2* (*Heat shock transcription factors A2*) (Zhang et al., 2015), *CBF3* (*C-repeat binding factor 3*) (Hu et al., 2013), *HKT1* (*High-Affinity K^+^ Transporter 1*) (Waters et al., 2013), and *GST* (*Glutathione S-transferase*) (Roxas et al., 1997) were conducted to confirm the validity of the RGs in this study. This work provide a list of suitable RGs for future gene expression studies in *S. psammophila*.

## Materials and Methods

### Plant Materials and Stress Treatments

Two-month-old seedlings of *S. psammophila* clones were cultured using Hoagland nutrient solution and grown in a growth chamber under long-day conditions (16 h light/8 h dark) at 25/22°C (day/night). Various tissues, including leaf (L), primary stem (PS), secondary stem (SS), root (R), female catkin (FC), and male catkin (MC) were collected from the *S. psammophila* seedlings. For stress treatments, the seedlings were subjected to abiotic stresses under heat (42°C), cold (4°C), salt (200 mM NaCl), and 22% (w/v) polyethylene glycol (PEG 6000)-simulated drought treatments. Leaves from individuals were harvested at six selected time points (0, 6, 12, 24, 48, and 96 h) during each stress treatments, frozen immediately in liquid nitrogen, and stored at -80°C for further analysis. Three biological replicates were performed in different pots for each treatment.

### Total RNA Isolation and cDNA Synthesis

RNA of different tissues and different stress treatments were extracted using the RNeasy Plant Mini Kit (Qiagen, Germany) according to the manufacturer’s instructions. RNA concentration were determined by NanoDrop8000 (Thermo Scientific, USA). Only the RNA sample with an absorbance ratio at OD260/280 between 1.9 and 2.2 and OD260/230 greater than 2.0 were used for further analysis and the RNA integrity were verified by 1.0% agarose gel-electrophoresis. First-strand cDNA synthesis was carried out with approximately 4 μg RNA using the SuperScript III reverse transcription kit (Invitrogen, USA) according to the manufacturer’s procedure, and the final product was diluted 40-fold for experiments.

### Selection of Candidate RGs and Primers Design

Here, we selected a total of 14 potential candidate RGs, including six novel RGs [*LIPL* (*Lipocalin-like gene*), *LTP* (*Seed storage/lipid transfer protein*), *ARF2* (*ADP-ribosylation factor 2*), *LTA4H* (*Leukotriene A-4 hydrolase homolog*), *VHAC* (*V-type proton ATPase subunit C*) and *OTU* (*OTU-like cysteine protease family protein*)] and eight traditional RGs [*TUB* (*Tubulin beta chain*), *HIS* (*Histone superfamily protein H3*), *ACT7* (*Actin 7*), *ACT11* (*Actin 11*), *UBC* (*Ubiquitin-conjugating enzyme E2*), *UBQ* (*Ubiquitin family*), *EF1*α (*Elongation factor-1 alpha*) and *Cpn60*β (*60-kDa chaperonin*β*-subunit*)] (**Table 1**) (Wang et al., 2014; Yim et al., 2015; Ma et al., 2016; Wu et al., 2016). Among of them, *ARF2*, *LTA4H*, *VHAC, and OTU* were selected as candidate RGs because they exhibited stable expression in the drought stress based on our transcript data (Unpublished). In addition, the traditional RGs and the two novel genes (*LTP* and *LTPL*) were chosen based on the previous studies. The sequence of these candidate RGs were obtained from transcript data. The Primers were designed using Primer3 software^1^ in the specific region of sequences, with melting temperatures of 58–62°C, 20–23 bp primer length and amplified product size of 150–250 bp. All the primer sequences in this study were listed in **Table 2**.

**Table 1:** The comprehensive details of the genes used for *Salix psammophila* RGs selection.

Gene abbreviation	Gene name	Gene accession in *S. purpurea*	Gene accession in *S. suchowensis*	Amplicon length (bp)	Amplification efficiency (%)	*R*^2^
*TUB*	Tubulin beta chain	SapurV1A.0612s0060.1	willow_GLEAN_10025465	190	105.57	0.9982
*HIS*	Histone superfamily protein H3	SapurV1A.0311s0010.1	willow_GLEAN_10003889	219	107.63	0.9993
*ACT7*	Actin 7	SapurV1A.0231s0320.1	willow_GLEAN_10001533	173	95.27	0.9946
*ACT11*	Actin 11	SapurV1A.0018s0700.1	willow_GLEAN_10010347	173	95.19	0.9947
*UBQ*	Ubiquitin family	SapurV1A.0014s0290.1	willow_GLEAN_10018914	207	91.98	0.9994
*EF1*α	Elongation factor-1 alpha	SapurV1A.0023s0300.1	willow_GLEAN_10019594	243	103.30	0.9996
*Cpn60*β	60-kDa chaperonin β-subunit	SapurV1A.0081s0600.1	willow_GLEAN_10013343	152	89.13	0.9995
*LIPL*	Lipocalin-like gene	SapurV1A.0347s0210.1	willow_GLEAN_10011584	174	93.69	0.9971
*LTP*	Seed storage/lipid transfer protein	SapurV1A.0214s0050.1	willow_GLEAN_10024899	164	93.68	0.9994
*ARF2*	ADP-ribosylation factor 2	SapurV1A.0014s0160.1	willow_GLEAN_10018905	246	91.18	0.9967
*LTA4H*	Leukotriene A-4 hydrolase homolog	SapurV1A.0023s0630.1	willow_GLEAN_10019570	156	112.13	0.9949
*UBC*	Ubiquitin-conjugating enzyme E2	SapurV1A.0237s0020.1	willow_GLEAN_10023705	183	97.25	0.9901
*VHAC*	V-type proton ATPase subunit C	SapurV1A.0123s0450.1	willow_GLEAN_10019233	189	119.11	0.9873
*OTU*	OTU-like cysteine protease family protein	SapurV1A.0615s0200.1	willow_GLEAN_10023705	187	115.76	0.9917

**Table 2:** The primer sequences used in this study.

Gene abbreviation	Forward primer sequence (5′–3′)	Reverse primer sequence (5′–3′)
*TUB*	ATGAGTGGAGTGACGTGCTG	CATCCCACATTTGCTGTGTC
*HIS*	AAGAGGCAGCTGAGGCATAC	TTCCACCGGTACAGCCTAAC
*ACT7*	GGTTTGCTGGTGATGATGCA	GCTGACAATACCGTGCTCAA
*ACT11*	TTCCCTTTATGCCAGTGGTC	AGCCACGCTCAGTCAAGATT
*UBQ*	AAGCCCAAGAAGATCAAGCA	ACCACCAGCCTTCTGGTAAA
*EF1*α	GATTTGAAGCGTGGGTTTGT	AGCATCTCCGTTCTTCAGGA
*Cpn60*β	TGCCAAAAATGCTGGTGTTA	GGCAACATCTGACCACCTTT
*LIPL*	CATTCTTGCCCATCATTCCT	CCTCTTCTTTGGCCTTCTCC
*LTP*	TCTGTGCTGTCATGCTTTCC	AGGGTGAGTGGAGTTCATGG
*ARF2*	TCTGATGGTGGGTCTCGATG	TCCACCACACGATCTCTGTC
*LTA4H*	TGGCTATTTCGTCCAGGTGT	CAGCAAACACTCTCTCTGCC
*UBC*	TGGCATCGAAACGGATCTTG	ACGGATAGTCTGGAGGAAAATGA
*VHAC*	GCCATTCGTGTCTTTGCAGA	TCCCAAGCCGAATATCCCTC
*OTU*	ATTGGTGAGGAGGTGCAAGA	CTGATCCCACCCCTTCATCA

*WOX1a*	CGATACAGCTCTTCGAGGGT	CCATCAACTTCAGCTGCCTC
*Hsf-A2*	GAGGGAACACATTGCTGCAA	CCACCTAATGCCACAGATGC
*CBF3*	TGCAGGGCGGAGAATATTCA	CCGAGTCCGCGAAATTAAGG
*HTK1*	TCCCAAGACCACAAACCACA	TTTCTTCCCTCGCAGACAGT
*GST*	GGATGTCTTACTGGCTGCCA	CGCGAGGCCTAGAAGAAAAC

### qRT-PCR Conditions

To verify the specificity of all the primer sets, PCR was performed using pooled cDNA as templates, and the PCR products were examined by 2% (w/v) agarose gel electrophoresis. The amplicons should appear as a single band with the correct size. qRT-PCR reactions were conducted on LightCycler^®^ 480 Real Time PCR System (Roche, Germany). The 20 μl reaction system contained 10 μl of SYBR Premix Ex Taq^TM^ (TaKaRa, China), 2 μl of cDNA template, 0.8 μl of each primer, and 6.4 μl ddH_2_O. The PCR conditions were conducted by the manufacturer (pre-incubation of 5 min at 95°C, amplification of 45 cycles of 95°C for 10 s, 60°C for 10 s and 72°C for 10 s, melting curve analysis by heating the PCR products from 65 to 95°C, finally by cooling at 40°C for 30 s). The final threshold cycle (*C*_t_) values were the mean of three values for each treatment and four technical replicates.

### Data Analysis of Gene Expression Stability

Expression levels of the 14 RGs in all samples were determined by their cycle threshold values (*C*_t_) which using the formula: 2^-Δ^*^C^*^t^, in which Δ*C*_t_ = each corresponding *C*_t_ value – minimum *C*_t_ value. Standard curves were generated using dilutions of the mixed cDNA template (1, 1/5, 1/25, 1/125, 1/625/, 1/3125). The correlation coefficients (*R*^2^ values) and slope (S) values can be obtained from standard curves. And the PCR efficiency (E) was derived from the expression [5^(1/-S)^ - 1] × 100% (Han et al., 2012).

Three software algorithms, geNorm (Vandesompele et al., 2002), NormFinder (Andersen et al., 2004), and BestKeeper (Pfaﬄ et al., 2004) were used to rank the stability of the selected RGs across all the experimental sets. The methods were performed according to Han et al. (2012). The RankAggreg package of R program v.3.2.3^2^ (Pihur et al., 2009) was used to merge the stability measurements obtained from the three software algorithms and establish a consensus rank of RGs according to the method described by Wang et al. (2014).

### Validation of Reference Genes

In order to validate the applicability of the identified RGs, the expression levels of five target genes, including one tissue-specific gene *WOX1a* and four stress-inducible genes, including *Hsf-A2* for heat stress, *CBF* for cold stress, *HKT1* for salt stress, and *GST* for drought stress were analyzed using the most and least stable RGs. The average *C*_t_ value was calculated from three biological replicates and four technical replicates and used for relative expression analyses. The relative gene expression data were calculated according to the 2^-ΔΔC_t_^ method and presented as fold change (Schmittgen and Livak, 2008).

## Results

### Verification of Amplicons, PCR Specificity, and Amplification Efficiency

A total of 14 candidate RGs were selected for qRT-PCR analysis. Gene name, the homologous gene accession in *S. purpurea* and *S. suchowensis*, amplicon length, amplification efficiency (E) and correlation coefficients (*R*^2^) were listed in **Table 1**. The amplified products were also further analyzed by 2% agarose gel electrophoresis and only one band of the expected size was detected, no primer dimers or non-specific amplification in each experiment (Supplementary Figure S1A). For PCR specificity detection, the presence of a single peak in the melting curve was obtained after amplification (Supplementary Figure S1B). Amplification efficiency of PCR reactions varied from 89.13% for *Cpn60*β to 119.11% for *VHAC*, and correlation coefficients of the standard curve ranged from 0.9873 for *VHAC* to 0.9996 for *EF1*α (**Table 1**).

### Expression Profiles of Candidate Reference Genes

**FIGURE 1:**
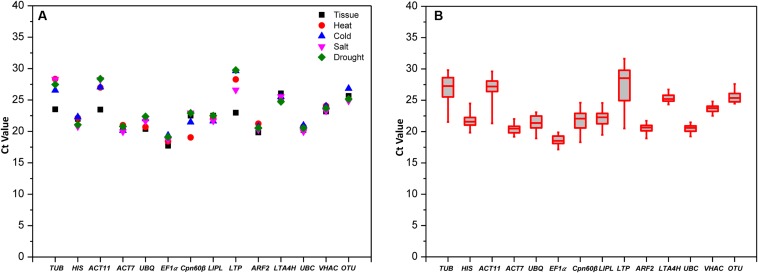
**qRT-PCR *C*_t_ values of the 14 candidate RGs in all experimental samples. (A)** Mean values in different treatments. **(B)** Box-whisker plot showing the *C*_t_ variation among 30 test samples. A line across the box depicts the median. In each box, the upper and lower edges indicate the 25th and 75th percentiles.

The RNA from different tissues and stress treatments were reverse transcribed into cDNA which was used as templates for qRT-PCR. The *C*_t_ values were obtained from each reaction with 14 primer pairs. Lower *C*_t_ values mean higher expression abundance, and the higher *C*_t_ values mean lower expression abundance. The average *C*_t_ values ranged from 17.72 to 29.76 in different tissues and four treatments (**Figure 1A**); *EF1*α had the highest expression level with *C*_t_ of 17.72 cycle, whereas *LTP* was the lowest expression abundance with *C*_t_ values as high as 29.76 cycle.

The *C*_t_ values data distribution of genes in all tested samples are shown as a box-plot in **Figure 1B**, the more narrow range of *C*_t_ values indicates that the gene is more stability in the conditions. The *C*_t_ data of *UBC* (*C*_t_ values from 19.22 to 21.48) with most concentration means it was the most stable gene in all the samples, and the followed stable genes were *VHAC* and *LTA4H* with *C*_t_ values from 22.52 to 24.81 and from 24.33 to 26.72, respectively. The greatest variation three genes were displayed by *LTP*, *ACT11*, and *TUB* (more than six *C*_t_ values), while the *C*_t_ values of *LTP* were the most scattered (*C*_t_ values from 20.50 to 31.64). These results indicated that the selected genes showed variable expression levels in different tissues and under four abiotic stresses, so it was necessary to screen out the best RG for target gene expression normalization.

### Statistical Analysis of qRT-PCR Data Using Three Bioinformatics Programs

In order to detect the stabilities of 14 candidate RGs, the three Excel-based software (NormFinder, geNorm, and BestKeeper) were used for statistical analysis. Data from different tissues and four treatments were analyzed separately, and then added together. The analysis results were as follows:

#### geNorm Analysis

**FIGURE 2:**
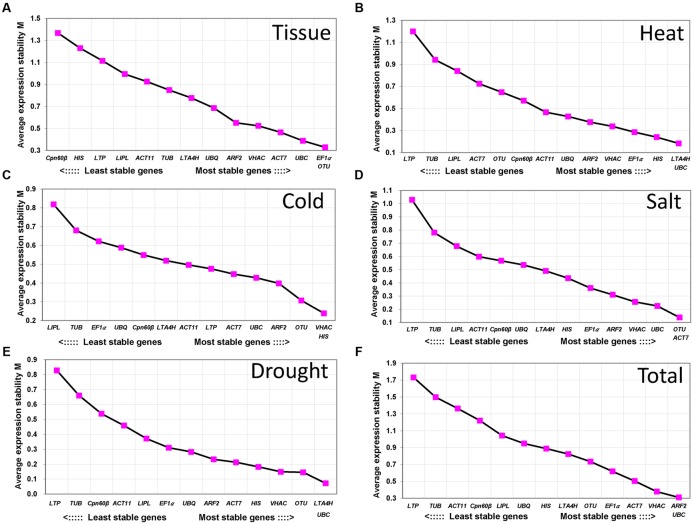
**Average expression stability values (*M*) and ranking of the candidate RGs calculated using geNorm.** *M*-value s and ranking of the candidate RGs in tissue **(A)**, heat stress **(B)**, cold stress **(C)**, salt stress **(D)**, drought stress **(E)**, and total sample **(F)**. A lower value of the average expression stability indicates more stable expression.

geNorm was used to rank the RGs by calculating gene expression stability value *M* based on the average pairwise expression ratio (Vandesompele et al., 2002). *M*-value is negatively correlated with gene stability which means the lower *M*-value of gene was considered as more stable gene. In this study, *M*-value setting 1.5 which recommends by geNorm Program was used to identify RGs with stable expression (Vandesompele et al., 2002). As shown in **Figure 2**, the value of all the 14 RGs were all below 1.5 in different tissues and treatments. In different tissues, *EF1*α, *OTU*, and *UBC* showed highly stable expression with the *M*-value below 0.5, whereas *Cpn60*β was the least stable gene with the *M*-value 1.38 (**Figure 2A**). In heat, cold and salt treatments, 8 of 14 genes were below 0.5 (**Figures 2B–D**). In drought treatment (**Figure 2E**), 11 of 14 genes were below 0.5. In heat and drought treatment, *UBC* and *LTA4H* were the most stable genes and the least stable gene was *LTP* (**Figures 2B,E**), while the *LTP* was also the least stable gene in salt treatment and the most stable gene were *OTU* and *ACT7* in salt treatment (**Figure 2D**). In cold treatment, *HIS* and *VHAC* were stably expressed (**Figure 2C**). Overall, all of the tested genes except *LTP* had low *M*-value s of less than 1.5, and *UBC* and *ARF2* were the most stable genes in all the samples (**Figure 2F**).

**FIGURE 3:**
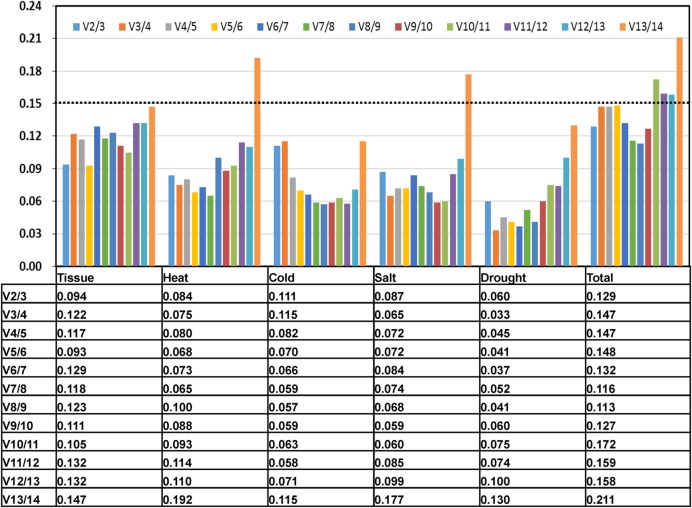
**PV to determine the optimal number of control genes for accurate normalization.** The pairwise variation (Vn/Vn+1) was analyzed between the NFn and NFn+1 using geNorm software, where n is the number of genes involved in the NF.

In some experiments, one RG can’t meet the requirement to effective normalize the gene expression and two or more RGs were required. In present study, the pairwise variation (Vn/Vn+1, n corresponds to the number of RGs) was calculated by geNorm program to evaluate the optimal number of the RGs required for accurate normalization (Vandesompele et al., 2002; Han et al., 2012). The value of Vn/Vn+1 generally set 0.15 as the threshold limit. When Vn/Vn+1 < 0.15, it means the number of RGs less than or equal to value of n is sufficient to use as RGs. In the subsets of tissues and treatments, all the V2/3 value were below 0.15, which suggested that two RGs would be enough used for normalization (**Figure 3**). Thus, the combined use of the two most stable RGs would be effective for normalizing gene expression studies.

#### NormFinder Analysis

NormFinder is also used to evaluate the stability of tested genes, which based on calculating the stability value for each gene (Andersen et al., 2004). The lowest stability value indicates the most stably expressed gene. The stability values of the tested genes are analyzed by NormFinder and the results were shown in **Table 3**. In different tissues, the most stable gene was *EF1*α (stability value 0.0560) and followed by *OTU* (0.1269), *UBC* (0.1364), and *ACT7* (0.2222). In heat treatment, the *UBC* (0.0647), *LTA4H* (0.0818), and *EF1*α (0.1113) had the most stable expression. In cold treatment, the three most stable genes were *HIS* (0.0416), *ARF2* (0.1061), and *OTU* (0.1189), while the most stable genes were *OTU* (0.0937), *VHAC* (0.1179), and *ACT7* (0.1466) in salt treatment. In cold and salt treatment, the expression levels of *TUB* was the most unstable. In drought treatment, *VHAC* (0.0293), *ACT7* (0.0579), and *ARF2* (0.0739) had stable expression. Finally, *UBC* (0.1272), *ARF2* (0.1542), and *VHAC* (0.1695) were the stable RGs, and the *LTP* (0.3978) was the least stable RG in total.

**Table 3:** Rankings of candidate RGs for normalization and their expression stability values calculated using the NormFinder program.

Rank	Tissue		Heat		Cold		Salt		Drought		Total	
	Gene	Stability	Gene	Stability	Gene	Stability	Gene	Stability	Gene	Stability	Gene	Stability
1	*EF1*α	0.0560	*UBC*	0.0647	*HIS*	0.0416	*OTU*	0.0937	*VHAC*	0.0293	*UBC*	0.1272
2	*OTU*	0.1269	*LTA4H*	0.0818	*ARF2*	0.1061	*VHAC*	0.1179	*ACT7*	0.0579	*ARF2*	0.1542
3	*UBC*	0.1364	*EF1*α	0.1113	*OTU*	0.1189	*ACT7*	0.1466	*ARF2*	0.0739	*VHAC*	0.1695
4	*ACT7*	0.2222	*HIS*	0.1263	*VHAC*	0.1245	*UBC*	0.1750	*OTU*	0.0866	*LTA4H*	0.1990
5	*ARF2*	0.2355	*VHAC*	0.1836	*UBC*	0.1493	*Cpn60*β	0.1906	*LTA4H*	0.0979	*OTU*	0.2029
6	*VHAC*	0.2377	*UBQ*	0.1974	*ACT7*	0.1822	*LTA4H*	0.2051	*UBC*	0.1117	*EF1*α	0.2310
7	*TUB*	0.2642	*ARF2*	0.2134	*ACT11*	0.1833	*HIS*	0.2086	*HIS*	0.1187	*ACT11*	0.2412
8	*ACT11*	0.3049	*ACT11*	0.2209	*Cpn60*β	0.2121	*ARF2*	0.2098	*EF1*α	0.1320	*HIS*	0.2516
9	*LTA4H*	0.3112	*ACT7*	0.2952	*EF1*α	0.2189	*UBQ*	0.2101	*UBQ*	0.1415	*ACT7*	0.2525
10	*LIPL*	0.3475	*Cpn60*β	0.2984	*UBQ*	0.2460	*ACT11*	0.2208	*ACT11*	0.2326	*UBQ*	0.2736
11	*LTP*	0.3567	*TUB*	0.3438	*LTP*	0.2478	*EF1*α	0.2503	*Cpn60*β	0.2414	*Cpn60*β	0.2930
12	*UBQ*	0.3608	*LIPL*	0.3579	*LTA4H*	0.2846	*LIPL*	0.2573	*TUB*	0.2828	*LIPL*	0.3211
13	*HIS*	0.4325	*OTU*	0.3631	*LIPL*	0.3377	*LTP*	0.3668	*LIPL*	0.2964	*TUB*	0.3431
14	*Cpn60*β	0.4593	*LTP*	0.4324	*TUB*	0.3879	*TUB*	0.4263	*LTP*	0.4837	*LTP*	0.3978

#### BestKeeper Analysis

BestKeeper program determines the stability ranking of the RGs based on calculating the coefficient of variance (CV) and the standard deviation (SD) of the *C*_t_ values (Pfaﬄ et al., 2004). The rankings of BestKeeper analysis were shown in the **Table 4**. For the different tissues, the *EF1*α (1.56 ± 0.28) was identified as the most stable gene in different tissues, *UBC* (0.55 ± 0.11) in heat treatment, *ARF2* (1.10 ± 0.23) in cold treatment, *OTU* (0.82 ± 0.20) in salt treatment, and *HIS* (0.40 ± 0.08) in drought treatment. In total, *VHAC* (1.92 ± 0.45) was the most stable gene and followed by *UBC* (2.29 ± 0.47), while the *LTP* (9.61 ± 2.64) was the least stable gene.

**Table 4:** Rankings of candidate RGs in order of their expression stability as calculated by BestKeeper.

Rank	Tissues			Heat			Cold			Salt			Drought			Total		
	Gene	*SD*	CV	Gene	*SD*	CV	Gene	*SD*	CV	Gene	*SD*	CV	Gene	*SD*	CV	Gene	*SD*	CV
1	*EF1*α	0.28	1.56	*UBC*	0.11	0.55	*ARF2*	0.23	1.10	*OTU*	0.20	0.82	*HIS*	0.08	0.40	*VHAC*	0.45	1.92
2	*OTU*	0.33	1.29	*EF1*α	0.16	0.87	*HIS*	0.24	1.09	*ACT7*	0.23	1.15	*LTA4H*	0.18	0.74	*UBC*	0.47	2.29
3	*UBC*	0.41	2.04	*HIS*	0.19	0.84	*VHAC*	0.26	1.10	*LTA4H*	0.27	1.06	*UBC*	0.22	1.06	*LTA4H*	0.50	1.98
4	*LTA4H*	0.44	1.67	*LTA4H*	0.19	0.78	*LTP*	0.27	0.92	*UBC*	0.35	1.76	*ACT7*	0.23	1.10	*ARF2*	0.58	2.82
5	*VHAC*	0.54	2.31	*UBQ*	0.21	1.03	*LTA4H*	0.30	1.18	*VHAC*	0.38	1.65	*VHAC*	0.31	1.29	*ACT7*	0.60	2.94
6	*UBQ*	0.68	3.33	*VHAC*	0.35	1.44	*UBC*	0.35	1.68	*ARF2*	0.41	2.00	*OTU*	0.32	1.29	*EF1*α	0.65	3.50
7	*ACT7*	0.70	3.42	*ACT11*	0.44	1.62	*OTU*	0.40	1.51	*EF1*α	0.42	2.24	*LIPL*	0.33	1.47	*OTU*	0.70	2.73
8	*ARF2*	0.72	3.62	*ARF2*	0.45	2.13	*Cpn60*β	0.44	2.06	*Cpn60*β	0.45	1.94	*EF1*α	0.36	1.87	*HIS*	0.78	3.59
9	*LIPL*	0.90	4.01	*Cpn60*β	0.54	2.82	*ACT11*	0.48	1.79	*UBQ*	0.53	2.45	*ARF2*	0.40	1.96	*UBQ*	0.80	3.72
10	*TUB*	0.94	4.02	*OTU*	0.64	2.54	*ACT7*	0.49	2.44	*ACT11*	0.59	2.07	*UBQ*	0.44	1.96	*LIPL*	0.99	4.47
11	*ACT11*	1.04	4.42	*ACT7*	0.81	3.88	*TUB*	0.52	1.94	*HIS*	0.66	3.16	*ACT11*	0.78	2.75	*Cpn60*β	1.41	6.48
12	*Cpn60*β	1.20	5.34	*LIPL*	0.94	4.25	*EF1*α	0.55	2.83	*TUB*	0.80	2.84	*Cpn60*β	0.82	3.56	*ACT11*	1.45	5.41
13	*LTP*	1.36	5.94	*TUB*	1.09	3.84	*UBQ*	0.57	2.58	*LIPL*	0.85	3.92	*TUB*	1.25	4.55	*TUB*	1.75	6.53
14	*HIS*	1.37	6.23	*LTP*	2.14	7.58	*LIPL*	1.53	7.08	*LTP*	1.99	7.47	*LTP*	1.30	4.38	*LTP*	2.64	9.61

### Consensus List and Validation of the Stability of S. psammophila Reference Genes

**FIGURE 4:**
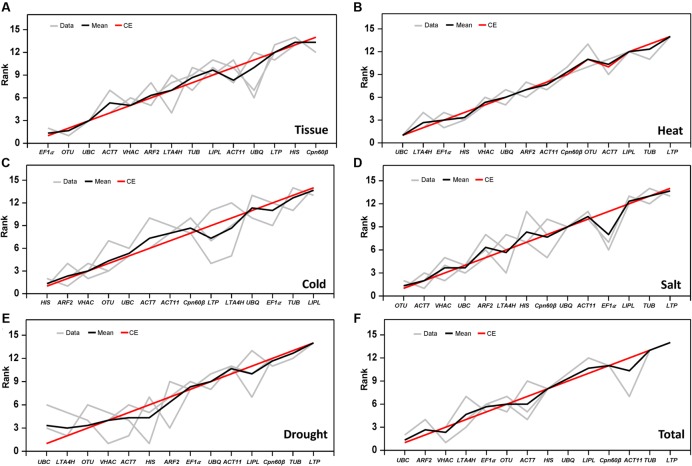
**Rank aggregation of gene lists using the Monte Carlo algorithm.** The solution of the rank aggregation in tissue **(A)**, heat stress **(B)**, cold stress **(C)**, salt stress **(D)**, drought stress **(E)**, and total sample **(F)** are shown in a plot in which genes are ordered based on their rank position according to each stability measurement (gray lines). The mean rank position of each gene is shown in black lines, as well as the model computed by the Monte Carlo algorithm (red lines).

In this study, three different software programs were used to analyze the stability of the 14 tested genes. However, different software use the different algorithm in gene ranking, so the results in ranking patterns showed little differences. In order to provide a comprehensive result, the RankAggreg software (Najafpanah et al., 2013) was used to rank an optimal lists of RGs. According to the analysis of RankAggreg (**Figure 4**; **Table 5**), the best RG for normalization was *EF1*α for different tissue types, *UBC* for heat and drought treatment, *HIS* for cold treatment, and *OTU* for salt treatment. When considering all the sample, the *UBC* was the best choice as the optimal RG (**Figures 4A–F**). The comprehensive ranking list by RankAggreg was shown in **Table 5**. Based on the optimal number of the RGs calculated by geNorm and the raking list by RankAggreg, the best combination of RGs in different subsets were shown in **Table 6**. The results shown that *UBC* and *LTA4H* were the best combination as RGs in heat and drought stress, while *UBC* and *ARF2* in total samples. In addition, the best combination included *EF1*α and *OTU* in tissues, *HIS* and *ARF2* in cold stress, *OTU* and *ACT7* in salt stress.

**Table 5:** Expression stability ranking of the 14 candidate RGs.

Method	1	2	3	4	5	6	7	8	9	10	11	12	13	14
**(A) Ranking order across different tissue types (Better-Good-Average)**					
geNorm	*OTU/EF1*α		*UBC*	*ACT7*	*VHAC*	*ARF2*	*UBQ*	*LTA4H*	*TUB*	*ACT11*	*LIPL*	*LTP*	*HIS*	*Cpn60*β
BestKeeper	*EF1*α	*OTU*	*UBC*	*LTA4H*	*VHAC*	*UBQ*	*ACT7*	*ARF2*	*LIPL*	*TUB*	*ACT11*	*Cpn60*β	*LTP*	*HIS*
NormFinder	*EF1*α	*OTU*	*UBC*	*ACT7*	*ARF2*	*VHAC*	*TUB*	*ACT11*	*LTA4H*	*LIPL*	*LTP*	*UBQ*	*HIS*	*Cpn60*β
RankAggreg	*EF1*α	*OTU*	*UBC*	*ACT7*	*VHAC*	*ARF2*	*LTA4H*	*TUB*	*LIPL*	*ACT11*	*UBQ*	*LTP*	*HIS*	*Cpn60*β
**(B) Ranking order under heat stress (Better-Good-Average)**					
geNorm	*UBC/LTA4H*		*HIS*	*EF1*α	*VHAC*	*ARF2*	*UBQ*	*ACT11*	*Cpn60*β	*OTU*	*ACT7*	*LIPL*	*TUB*	*LTP*
BestKeeper	*UBC*	*EF1*α	*HIS*	*LTA4H*	*UBQ*	*VHAC*	*ACT11*	*ARF2*	*Cpn60*β	*OTU*	*ACT7*	*LIPL*	*TUB*	*LTP*
NormFinder	*UBC*	*LTA4H*	*EF1*α	*HIS*	*VHAC*	*UBQ*	*ARF2*	*ACT11*	*ACT7*	*Cpn60*β	*TUB*	*LIPL*	*OTU*	*LTP*
RankAggreg	*UBC*	*LTA4H*	*EF1*α	*HIS*	*VHAC*	*UBQ*	*ARF2*	*ACT11*	*Cpn60*β	*OTU*	*ACT7*	*LIPL*	*TUB*	*LTP*
**(C) Ranking order under cold stress (Better-Good-Average)**					
geNorm	*HIS/VHAC*		*OTU*	*ARF2*	*UBC*	*ACT7*	*LTP*	*ACT11*	*LTA4H*	*Cpn60*β	*UBQ*	*EF1*α	*TUB*	*LIPL*
BestKeeper	*ARF2*	*HIS*	*VHAC*	*LTP*	*LTA4H*	*UBC*	*OTU*	*Cpn60*β	*ACT11*	*ACT7*	*TUB*	*EF1*α	*UBQ*	*LIPL*
NormFinder	*HIS*	*ARF2*	*OTU*	*VHAC*	*UBC*	*ACT7*	*ACT11*	*Cpn60*β	*EF1*α	*UBQ*	*LTP*	*LTA4H*	*LIPL*	*TUB*
RankAggreg	*HIS*	*ARF2*	*VHAC*	*OTU*	*UBC*	*ACT7*	*ACT11*	*Cpn60*β	*LTP*	*LTA4H*	*UBQ*	*EF1*α	*TUB*	*LIPL*
**(D) Ranking order under salt stress (Better-Good-Average)**					
geNorm	*ACT7/OTU*		*UBC*	*VHAC*	*ARF2*	*EF1*α	*HIS*	*LTA4H*	*UBQ*	*Cpn60*β	*ACT11*	*LIPL*	*TUB*	*LTP*
BestKeeper	*OTU*	*ACT7*	*LTA4H*	*UBC*	*VHAC*	*ARF2*	*EF1*α	*Cpn60*β	*UBQ*	*ACT11*	*HIS*	*TUB*	*LIPL*	*LTP*
NormFinder	*OTU*	*VHAC*	*ACT7*	*UBC*	*Cpn60*β	*LTA4H*	*HIS*	*ARF2*	*UBQ*	*ACT11*	*EF1*α	*LIPL*	*LTP*	*TUB*
RankAggreg	*OTU*	*ACT7*	*VHAC*	*UBC*	*ARF2*	*LTA4H*	*HIS*	*Cpn60*β	*UBQ*	*ACT11*	*EF1*α	*LIPL*	*TUB*	*LTP*
**(E) Ranking order under drought stress (Better-Good-Average)**					
geNorm	*UBC/LTA4H*		*OTU*	*VHAC*	*HIS*	*ACT7*	*ARF2*	*UBQ*	*EF1*α	*LIPL*	*ACT11*	*Cpn60*β	*TUB*	*LTP*
BestKeeper	*HIS*	*LTA4H*	*UBC*	*ACT7*	*VHAC*	*OTU*	*LIPL*	*EF1*α	*ARF2*	*UBQ*	*ACT11*	*Cpn60*β	*TUB*	*LTP*
NormFinder	*VHAC*	*ACT7*	*ARF2*	*OTU*	*LTA4H*	*UBC*	*HIS*	*EF1*α	*UBQ*	*ACT11*	*Cpn60*β	*TUB*	*LIPL*	*LTP*
RankAggreg	*UBC*	*LTA4H*	*OTU*	*VHAC*	*ACT7*	*HIS*	*ARF2*	*EF1*α	*UBQ*	*ACT11*	*LIPL*	*Cpn60*β	*TUB*	*LTP*
**(F) Ranking order under total samples (Better-Good-Average)**					
geNorm	*UBC/ARF2*		*VHAC*	*ACT7*	*EF1*α	*OTU*	*LTA4H*	*HIS*	*UBQ*	*LIPL*	*Cpn60*β	*ACT11*	*TUB*	*LTP*
BestKeeper	*VHAC*	*UBC*	*LTA4H*	*ARF2*	*ACT7*	*EF1*α	*OTU*	*HIS*	*UBQ*	*LIPL*	*Cpn60*β	*ACT11*	*TUB*	*LTP*
NormFinder	*UBC*	*ARF2*	*VHAC*	*LTA4H*	*OTU*	*EF1*α	*ACT11*	*HIS*	*ACT7*	*UBQ*	*Cpn60*β	*LIPL*	*TUB*	*LTP*
RankAggreg	*UBC*	*ARF2*	*VHAC*	*LTA4H*	*EF1*α	*OTU*	*ACT7*	*HIS*	*UBQ*	*LIPL*	*Cpn60*β	*ACT11*	*TUB*	*LTP*

**Table 6:** Best combination of RGs based on the geNorm and RankAggreg programs.

Experimental conditions					
Tissues	Heat	Cold	Salt	Drought	Total
*EF1*α	*UBC*	*HIS*	*OTU*	*UBC*	*UBC*
*OTU*	*LTA4H*	*ARF2*	*ACT7*	*LTA4H*	*ARF2*

### Reference Gene Validation

**FIGURE 5:**
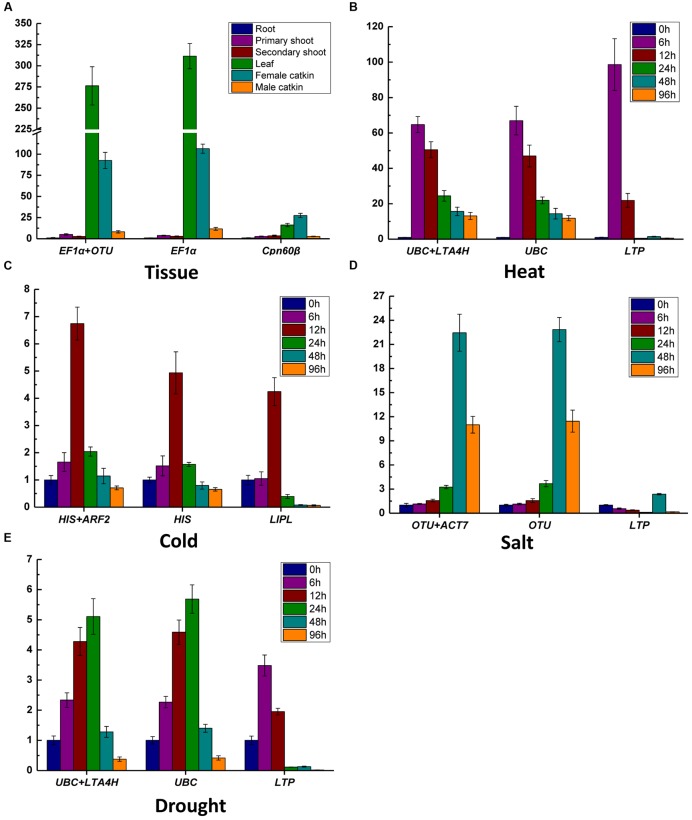
**Validation of the identified reference genes.** The relative expression levels of *WOX1a* **(A)***, Hsf-A2* **(B)***, CBF3* **(C)**, *HKT1* **(D)**, and *GST* **(E)** were normalized by the most stably RGs and the least stable RGs in the different samples. The transcript abundance of *WOX1a* in root was used as control in **(A)**, and the transcript abundances of four genes (*Hsf-A2*, *CBF3*, *HKT1*, and *GST*) at 0 h were set to be the control in **(B–E)**, individually. Values shown are relative expression levels ±SE (*n* = 3 experiments).

In order to assess the effect of the selected RG on the outcome of a practical experiment, the transcription levels of target genes were evaluated using the most and least stable RGs (**Figure 5**). *WOX1a* was reported high expression abundance in leaf in *A. thaliana* and poplar (Nakata et al., 2012; Liu M. et al., 2014), and it was detected for different tissues. The expression of *WOX1a* was high expression abundance in leaf when using both of *EF1*α and *OTU* or only *EF1*α as RG, but high expressed in female catkin when normalized by the least stable gene *Cpn60*β (**Figure 5A**). The expression level of *Hsf-A2* (Zhang et al., 2015), *CBF3* (Hu et al., 2013), *HKT1* (Waters et al., 2013), *GST* (Roxas et al., 1997) were detected in heat, cold, salt, and drought stress, respectively. For example, in drought stress treatment, when the two stable RGs *UBC* and *LTA4H* or only *UBC* were/was used for normalization, the expression levels of *HKT1* gradually increased from 0 h to 24 h and reached maximum at 24 h and subsequently decreased at 48 and 96 h (**Figure 5E**). However, when *LTP* was used as RG for normalization, different expression patterns were generated that the expression levels of *HKT1* peaked at 6 h and decreased at 12 h. Meantime, the expression pattern of the other target genes are also obvious different in the expression tendency when different RGs were used to normalize the data in heat, cold and salt (**Figures 5B–D**). These results confirmed the importance of using suitable gene as RG in different experiments.

## Discussion

Presently, qRT-PCR as a revolutionary technology has been widely used in detecting the gene expression profiling (Bustin, 2002; Radonić et al., 2004). But unsuitable RGs which used to normalize the experimental data in qRT-PCR will cause the deviation of results. So the aim of this study was looking for the ideal RGs with a stable expression levels in various expression conditions in *S. psammophila*.

Here, 14 candidate RGs including eight traditional genes and six novel genes were screened across different tissue types as well as under four abiotic stress treatments. *UBC*, *VHAC* and *LTA4H* with narrow *C*_t_ values were more stable, while the house-keeping genes *TUB* and *ACT11* with *C*_t_ values were scattered and reach to more than six cycles showing great expression variation (**Figure 1**). It was consistent with the recent studies that the expression of traditional RGs were not always stable in some experiments (Schmittgen and Zakrajsek, 2000; Jain et al., 2006; Hong et al., 2008; Wang et al., 2014; Kong et al., 2016; Xu et al., 2016).

The optimal number of stable RGs depends on accurate calculation following certain experimental conditions. In our study, two stable RGs can ensure the accuracy to detect the genes expression in different tissues and four stress experiment followed the analysis of geNorm program (**Figure 3**). While in pearl millet, two RGs were enough for individual stress and developmental tissues and three RGs were need in multiple stress (Shivhare and Lata, 2016). In watermelon, nine RGs were required for normalization of target genes for organ and tissue, but two RGs were enough for biotic stress (Kong et al., 2014a). In tung tree, two RGs would be sufficient to normalize the expression of the target gene in different tissues, but at least five RGs are required to ensure the accuracy of the expression in all the sample (Han et al., 2012). It also proved five RGs for salicylic acid (SA) stress and three RGs for heat and gibberellin (GA) stress conditions in carrot (Tian C. et al., 2015). Four RGs were needed for data normalization in melon leaf and root (Kong et al., 2014b), and five RGs were needed for data normalization in melon fruits (Kong et al., 2016).

Three different software packages including geNorm, NormFinder, and BestKeeper were employed to evaluate gene expression stability in our analysis. The different calculation algorithms of the three software lead to the divergence in the gene ranking, especially in the top ranked gene (**Table 5**). For instance, under drought stress *UBC* and *LTA4H* were identified as the best RG by geNorm, while *HIS* was the best RG predicated by BestKeeper, and *VHAC* was identified as the most ideal RG by NormFinder program. The difference ranking by three program was also presented in other studies (Kong et al., 2014a; Kanakachari et al., 2016; Li et al., 2016; Wu et al., 2016). In order to summarize the ranking of our dataset analysis, the RankAggreg software was used to merge the data (Wang et al., 2014). The best combination of RGs was get based on the analysis of geNorm and RankAggreg (**Figure 4**; **Tables 5** and **6**). Thereinto, three house-keeping genes (*UBC*, *EF1*α, and *HIS*) were the good choice as RGs in this study. These genes are also often considered as reliable RGs in other studies. For example, *UBC* was one of the most stable genes in salt and cold stress in *Lycoris aurea* (Ma et al., 2016) and it also showed high stability in the different cultivars of Tree Peony (Li et al., 2016). *EF1*α was one of the best RG which has been confirmed in pearl millet (Shivhare and Lata, 2016), rice (Jain et al., 2006), *Petunia hybrid* (Mallona et al., 2010), and *P. euphratica* (Wang et al., 2014).

In the present study, six new RGs (*OTU*, *LTA4H*, *ARF2*, *VHAC*, *LIPL*, and *LTP*) were detected as the candidate RGs. Based on the results, we found *OTU*, *LTA4H*, *ARF2*, and *VHAC* were the ideal choice for RGs in special experimental condition. Among of them*, OTU* gene family exist in various organisms, and involved in the de-ubiquitination signaling (Frias-Staheli et al., 2007), but it is the first time to be reported as a stable RG in plant study. *LTA4H* is one of the best choice under heat stress and drought stress. It is a bifunctional zinc enzyme which generates the inflammatory mediator leukotriene B4 (LTB4) and involved in many defense mechanism in human, like as inflammation, immune responses and others (Haeggstrom, 2000; Thunnissen et al., 2001), The structure and characterization also were studied in *Saccharomyces cerevisiae* (Kull et al., 1999), but the understanding of the function of *LTA4H* in plant is limited. ARF proteins were defined by their ability to act as cofactors in the cholera toxin-catalyzed ADP-ribosylation of G proteins and play role in membrane transport, maintenance of organelle integrity, and the activation of phospholipase D (Bhamidipati et al., 2000). *VHAC* is part of V-type proton ATPase (Beyenbach and Wieczorek, 2006) and plays as a regulatory linker protein between the V-ATPase and the actin-based cytoskeleton (Marshansky and Futai, 2008). And the recent study reported that *VHAC* involved in the drought stress (Gao et al., 2011). These four novel genes were the suitable RGs because of the stable expression in different experiment conditions, but the limited information about these genes were known in plant and the further study will be necessary. In our study, other two novel genes (*LIPL* and *LTP*) were studied as the candidate RGs. These two genes had been studied as RGs in *P. euphratica* in which *LTP* was the most stable gene during cold, but not for other treatment (Wang et al., 2014). It also revealed the RGs was different in species and experimental conditions.

To validate the suitability of the RGs we identified, the expression of *WOX1a, Hsf-A2, CBF3*, *HKT1*, and *GST* were detected in various tissues and abiotic stress using different RGs. The date once again demonstrated that RGs play a key role in normalizing the data of the qRT-PCR, and the inappropriate RGs may lead to incorrect results for the target genes.

## Conclusion

The expression stability of 14 candidate RGs were detected across tissue types and under four abiotic stress treatments using four software in *S. psammophila*. And the optimum RG and the best combination of RGs were identified in different experiment conditions. To the best of our knowledge, it is the first study to identify the suitable RGs for normalizing the gene expression studies using qRT-PCR in *S. psammophila*. And it provides new information which will be useful to analyze the expression profiles and function of target genes involved in the development and stress tolerance in *S. psammophila*.

## Author Contributions

JL and HJ performed most of the experiments and wrote the manuscript. XH designed the experiments and edited the manuscript. JZ and PS helped in data collection, sample preparation, and RNA extraction. ML and JH coordinated the project, conceived, and designed the experiments. All authors read and approved the final manuscript.

## Conflict of Interest Statement

The authors declare that the research was conducted in the absence of any commercial or financial relationships that could be construed as a potential conflict of interest.

## Footnotes

1 http://bioinfo.ut.ee/primer3/

2 http://www.r-project.org/
