# Supplementary material for: Selection of Reliable Reference Genes for Gene Expression Analysis under Abiotic Stresses in the Desert Biomass Willow, Salix psammophila
Source: Front Plant Sci. 2016 Oct 5;7:1505. doi: 10.3389/fpls.2016.01505 (PMC5050224; doi:10.3389/fpls.2016.01505)
Supplement: Supplementary file 1 [file Presentation_1.pdf]

Supplementary Material

Selection of reliable reference genes for gene expression analysis under abiotic stresses in the desert biomass willow, *Salix psammophila*

Jianbo Li[1†], Huixia Jia[1, 2†], Xiaojiao Han[1], Jin Zhang[1], Pei Sun[1], Mengzhu Lu[1, 2], Jianjun Hu[1, 2*]

[1] State Key Laboratory of Tree Genetics and Breeding, Key Laboratory of Tree Breeding and Cultivation of the State Forestry Administration, Research Institute of Forestry, Chinese Academy of Forestry, Beijing 100091, China

[2] Collaborative Innovation Center of Sustainable Forestry in Southern China, Nanjing Forestry University, Nanjing, 210037, China

[*] Corresponding author at: State Key Laboratory of Tree Genetics and Breeding, Key Laboratory of Tree Breeding and Cultivation of the State Forestry Administration, Research Institute of Forestry, Chinese Academy of Forestry, Beijing 100091, China. Tel.: +86 10 62888862. E-mail addresses: hujj@caf.ac.cn (J. Hu)

[†]These authors have contributed equally to this work.

Supplementary Figures

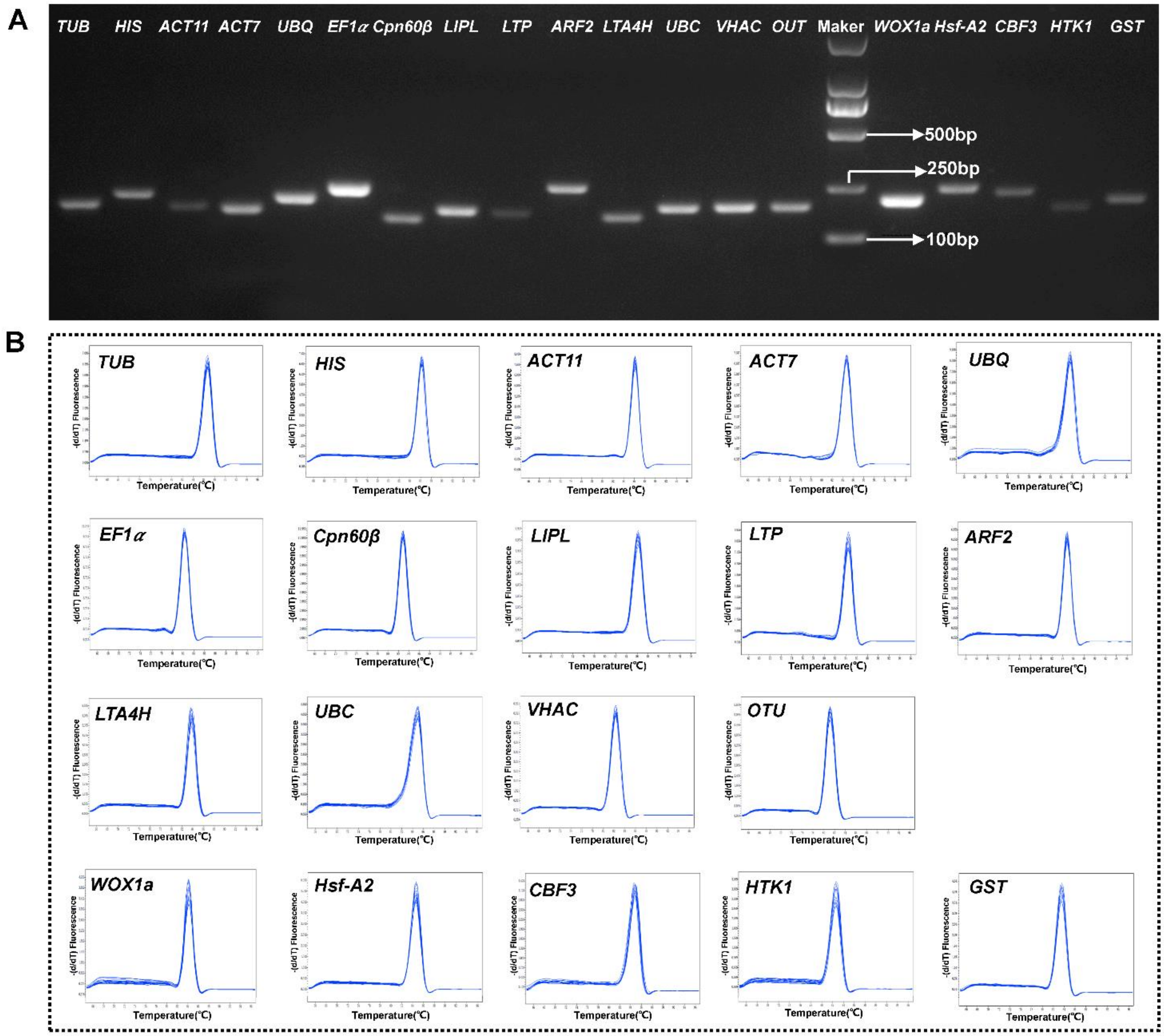

Supplementary Figure S1. Gene specificity and amplicon size. (A) 2% Agarose gel electrophoresis showing amplification of a specific PCR product and size of the expected size for each gene. (B) Melting curves of 14 reference genes and five target genes showing single peaks.
